# Unsupervised Learning-Based Anomaly Detection for Bridge Structural Health Monitoring: Identifying Deviations from Normal Structural Behaviour

**DOI:** 10.3390/s26020561

**Published:** 2026-01-14

**Authors:** Jabez Nesackon Abraham, Minh Q. Tran, Jerusha Samuel Jayaraj, Jose C. Matos, Maria Rosa Valluzzi, Son N. Dang

**Affiliations:** 1 University of Minho, ISISE, ARISE, Department of Civil Engineering, 4800-058 Guimarães, Portugal; 2 Department of Information Systems, University of Minho, 4800-058 Guimarães, Portugal; 3 Department of Cultural Heritage, University of Padova, Piazza Capitaniato 7, 35139 Padova, Italy

**Keywords:** SHM, unsupervised learning, ensemble fusion, principal component analysis, autoencoder, anomaly detection, adaptive weighting, Z24 dataset

## Abstract

Structural Health Monitoring (SHM) of large-scale civil infrastructure is essential to ensure safety, minimise maintenance costs, and support informed decision-making. Unsupervised anomaly detection has emerged as a powerful tool for identifying deviations in structural behaviour without requiring labelled damage data. The study initially reproduces and implements a state-of-the-art methodology that combines local density estimation through the Cumulative Distance Participation Factor (CDPF) with Semi-parametric Extreme Value Theory (SEVT) for thresholding, which serves as an essential baseline reference for establishing normal structural behaviour and for benchmarking the performance of the proposed anomaly detection framework. Using modal frequencies extracted via Stochastic Subspace Identification from the Z24 bridge dataset, the baseline method effectively identifies structural anomalies caused by progressive damage scenarios. However, its performance is constrained when dealing with subtle or non-linear deviations. To address this limitation, we introduce an innovative ensemble anomaly detection framework that integrates two complementary unsupervised methods: Principal Component Analysis (PCA) and Autoencoder (AE) are dimensionality reduction methods used for anomaly detection. PCA captures linear patterns using variance, while AE learns non-linear representations through data reconstruction. By leveraging the strengths of these techniques, the ensemble achieves improved sensitivity, reliability, and interpretability in anomaly detection. A comprehensive comparison with the baseline approach demonstrates that the proposed ensemble not only captures anomalies more reliably but also provides improved stability to environmental and operational variability. These findings highlight the potential of ensemble-based unsupervised methods for advancing SHM practices.

## 1. Introduction

The assessment of civil infrastructure, particularly bridges, has become increasingly vital as the world’s structural assets age and traffic demands intensify. Traditional methods, based on visual inspection, enable the assessment of the external condition of structures [1]. However, assessments of potential damage within structures are often difficult to detect [2]. In addition, traditional solutions such as visual inspection are time-consuming and resource intensive. This calls for the development of highly reliable structural health monitoring (SHM) systems. With the development of computer science and ultra-sensitive sensors, SHM systems now allow for automated, data-driven anomaly detection. SHM systems use continuous data streams from vibration or deformation sensors and analyse the responses to monitor structural health [3]. By continuously tracking the response of structures, SHM enables early detection of damage, thereby reducing maintenance costs and preventing catastrophic failures [4,5]. Over the past two decades, numerous data-driven approaches have been proposed to automate damage detection, ranging from supervised learning with labelled damage data to fully unsupervised anomaly detection frameworks [6].

However, in most real-world SHM scenarios, labelled damage data are extremely scarce or unavailable [7,8]. Structures typically remain in healthy conditions for long periods, and controlled damage experiments are costly and often infeasible. As a result, traditional supervised classifiers cannot be reliably trained [9,10]. Recent research has therefore shifted toward unsupervised anomaly detection. The requirement is only undamaged-state data for model training. In parallel, physics-based and hybrid approaches have also gained attention, integrating first-principles models and uncertainty quantification to improve damage diagnosis. For example, transmissibility-based methods combined with probabilistic distance measures have been shown to capture structural changes while accounting for model uncertainty, offering a complementary perspective to data-driven anomaly detection approaches [11].

Over the past two decades, SHM research has advanced from classical damage detection techniques to modern machine-learning and physics-informed paradigms [7,10,12] While supervised learning and transfer learning can achieve strong performance when representative labelled damage data exist [8], real-world bridge monitoring rarely provides such data: structures typically remain healthy for long periods, and controlled damage experiments are costly and often infeasible. This practical constraint has shifted increasing attention toward unsupervised anomaly detection, where models are trained using only undamaged-state data and anomalies are identified as departures from the learned normal behaviour [9,10]. In parallel, probabilistic and Bayesian perspectives have gained prominence for handling uncertainty in long-term monitoring. Bayesian approaches have been utilized for condition assessment in field applications [13,14], and recent work in modal identification has demonstrated that Bayesian spectral decomposition can estimate modal parameters with quantified uncertainty under ambient vibration, thereby improving robustness to noise and modelling errors [15]. These trends underscore the central challenge of long-term SHM: reliable anomaly detection under environmental and operational variability, with limited or no labelled damage data.

A substantial body of unsupervised SHM research relies on distance- and statistics-based deviations. Mahalanobis-distance monitoring, including ensemble formulations, has been used for anomaly detection and damage localisation under non-stationary signals [16,17]. Other pattern-recognition approaches include divergence-based novelty detection [18], dynamic time warping for ambient and non-stationary responses [19], and distance measures such as optimal sub-pattern assignment for substructural diagnosis [20]. Dimensionality-reduction and multivariate monitoring methods have also been used to create compact health indicators [21,22], while clustering-based outlier detection has been applied to long-term bridge monitoring, including spectral clustering and clustering-driven threshold estimation under environmental variability [23,24,25,26]. These approaches are often interpretable and computationally efficient, yet they can be sensitive to non-stationary behaviour and non-Gaussian distributions induced by varying temperature, traffic, and operational conditions [13,14,17,18].

To mitigate environmental variability and improve robustness, linear subspace methods—most notably PCA and its variants—have been widely adopted to characterise healthy behaviour and separate dominant environmental trends from damage-sensitive deviations [27,28]. Temperature-driven MPCA has been proposed to address thermal effects [29], PCA combined with time-series modelling has supported long-term monitoring of large structures [30], and moving-PCA strategies have been reported for highway bridges operating under changing conditions [31]. However, PCA-based models are inherently limited in capturing nonlinear interactions and manifold distortions frequently observed in real structures. This limitation has encouraged reconstruction-based learning, especially autoencoders, which can learn nonlinear representations of healthy dynamics and flag anomalies through reconstruction errors [32,33,34,35,36,37,38]. Comparative investigations have demonstrated the potential of autoencoders for structural damage identification [39], and their performance has been shown to depend on monitoring configurations such as measurement intervals in uncontrolled SHM [40,41]. For the Z24 bridge, stacked autoencoders have been employed to extract damage-sensitive features for unsupervised detection, typically relying on a single reconstruction error indicator [42]. Nevertheless, autoencoders may overlook anomalies that remain close to the learned manifold, or that induce only subtle reconstruction deviations under certain damage–environment interactions [39,41]. Recent hybrid efforts suggest combining linear and nonlinear ideas, for example, by enhancing PCA with deep learning components to better represent nonlinear residual behaviour [43] or by using variational autoencoders for representation learning in SHM contexts [44]. Yet such approaches commonly treat linear and nonlinear components sequentially or as enhancements within a single model family, rather than integrating complementary linear and nonlinear detectors within a unified unsupervised ensemble explicitly designed for robust long-term anomaly detection [43].

Ensemble-based anomaly detection has been explored as a route to improved reliability, but practical SHM ensembles often rely on static fusion rules because labelled damage data are unavailable to train complex fusion layers. Distance-metric ensembles have been reported for SHM monitoring under varying conditions [16], and the broader data-fusion literature in SHM documents a wide range of fusion strategies and their trade-offs [45]. While more dynamic directions—such as reinforcement-learning-driven ensembles for SHM [46] and unsupervised ensembles of streaming anomaly detectors [47] are emerging, there remains a gap in principled, performance-driven weighting that can be calibrated using only undamaged-state data and that adapts detector contributions according to their stability under environmental and operational variability. Threshold selection represents another persistent limitation. EVT provides a statistically grounded basis for modelling tail behaviour in streaming anomaly detection [48], and EVT-type strategies have been applied to online anomaly detection in long-term SHM [49]. However, existing EVT-based SHM implementations largely focus on modeling the tail of a single anomaly indicator and do not explicitly account for the heterogeneous, non-Gaussian tail behavior that can arise when multiple heterogeneous anomaly scores are fused. This limits the direct applicability of single-detector EVT thresholding to ensemble anomaly detection in SHM [50,51].

Motivated by these gaps, this study proposes an ensemble-based unsupervised anomaly detection framework for modal-feature-based SHM that integrates complementary linear and nonlinear perspectives and provides statistically principled decision-making under long-term variability. The framework combines PCA-based subspace monitoring and autoencoder-based nonlinear reconstruction and fuses their heterogeneous anomaly scores through an adaptive weighting mechanism calibrated from undamaged-state validation statistics, avoiding heuristic or fixed fusion rules. To enable robust alarm decisions, a Weighted Semi-parametric Extreme Value Theory (SEVT) thresholding strategy is introduced to explicitly model the tail behaviour of the fused anomaly score distribution, addressing heterogeneity induced by score fusion beyond conventional single-indicator EVT formulations. The approach is validated on the Z24 bridge dataset [52], where long-term monitoring data exhibit pronounced environmental influences and documented damage events [53]. The main contributions are:

(i) A unified unsupervised ensemble that jointly leverages PCA and autoencoders to exploit complementary linear and nonlinear sensitivities [31,39,42].
(ii) An adaptive, performance-based fusion mechanism that adjusts detector contributions using healthy-state stability without requiring labelled damage data [45,46,47].
(iii) A weighted SEVT thresholding technique that models the heterogeneous tail behaviour of fused anomaly scores to provide statistically rigorous, environment-adaptive thresholds [12,48,49,54].

The remainder of this paper is organised as follows. Section 2 presents the proposed methodology, which includes feature extraction, ensemble design, and threshold modelling. Section 3 describes the experimental setup and results. Section 4 discusses findings and implications, and Section 5 concludes the paper.

## 2. Methodology

### 2.1. Overview of the Architectural Flow

**Figure 1:**
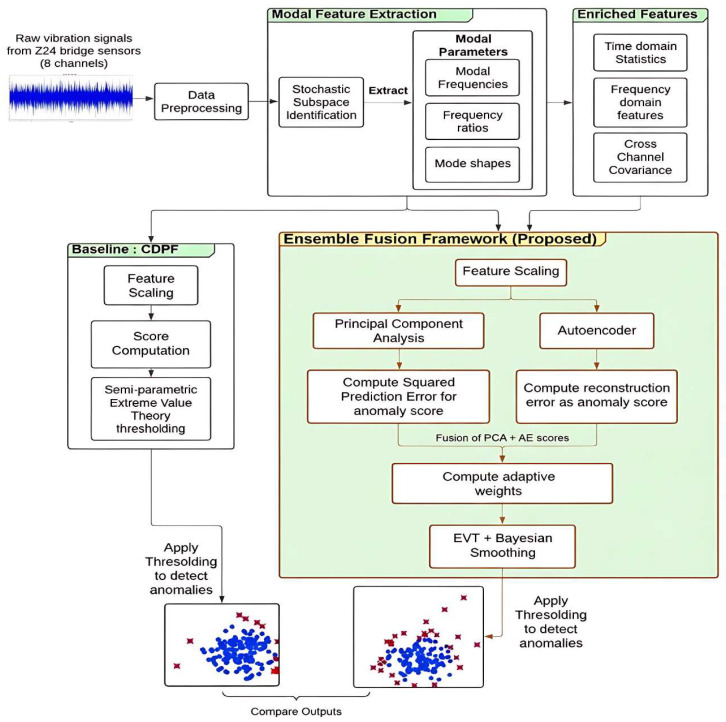
Comparative flow of the baseline CDPF–SEVT and the proposed PCA–AE Ensemble Fusion Framework.

The proposed methodology, as depicted in Figure 1, begins with the acquisition of raw vibration data from the well-known Z24 Bridge, a benchmark structure extensively used in structural health monitoring studies. The dataset comprises synchronised acceleration measurements obtained from eight strategically placed sensors that continuously capture the bridge’s dynamic response to varying environmental and operational loads. Prior to analysis, the raw acceleration signals undergo pre-processing to eliminate low-frequency drift, electrical noise, and channel misalignments, ensuring signal integrity. The cleaned signals are then divided into fixed-length segments, each representing a quasi-stationary state of the structure. This segmentation facilitates the temporal tracking of dynamic behaviour across multiple loading and environmental conditions, thereby enabling a detailed assessment of structural evolution and potential damage progression over time.

### 2.2. Baseline: CDPF with SEVT Thresholding

The baseline CDPF-SEVT anomaly detection framework is formulated to quantify deviations in the dynamic response of structural systems by integrating three complementary stages: Stochastic Subspace Identification (SSI) for modal feature extraction, CDPF for deviation scoring, and SEVT for adaptive threshold calibration. This combination yields a fully unsupervised, interpretable pipeline that forms a statistically rigorous baseline for evaluating advanced non-linear and data-fusion-based damage detection models.

The CDPF component quantifies each segment’s deviation from the nominal (undamaged) structural manifold in the transformed feature space. Despite its conceptual simplicity, the CDPF score effectively captures macro-level shifts in modal energy distributions caused by physical deterioration, material degradation, or structural boundary variations [34]. To separate normal from anomalous states, an adaptive statistical threshold is established using the Peaks-Over-Threshold (POT) formulation of the Generalised Pareto Distribution (GPD) [26]. The CDPF–SEVT baseline, while statistically principled and computationally efficient, exhibits few intrinsic limitations that constrain its applicability to complex structural behaviours. Its reliance on linear SSI-derived modal features limits its sensitivity to non-linear phenomena, such as modal coupling or frequency bifurcation, which are often observed in advanced stages of structural degradation. The assumption of signal stationarity during Power Spectral Density (PSD) estimation further limits consistency under varying environmental or operational conditions, where time-varying excitations can distort modal estimates. Additionally, averaging spectral information across channels suppresses spatial mode-shape variations, masking localised damage signatures.

These limitations collectively motivate the development of a more expressive ensemble fusion framework that integrates complementary representations to overcome the inherent linearity and stationarity constraints of the baseline CDPF-SEVT. This ensemble fusion enables the joint capture of both linear and non-linear feature correlations, enhances sensitivity to subtle structural deviations, and provides greater resilience against noise and environmental variability, thereby extending the applicability of anomaly detection to more complex and heterogeneous structural health monitoring scenarios.

### 2.3. Ensemble Fusion Framework

The proposed Ensemble Fusion (PCA–AE) Framework, shown in Figure 1, constitutes an advanced hybrid architecture for vibration-based structural health monitoring. Its purpose is to identify abnormal structural states, such as stiffness loss, joint degradation, or boundary loosening, by integrating linear and non-linear feature learning mechanisms. To address this challenge, the Ensemble Fusion Framework introduces a multi-stage data-driven anomaly detection pipeline that synergistically combines multi-domain feature extraction, dual-path modelling (PCA and AE), and adaptive statistical thresholding within a unified learning paradigm. The approach extends the baseline CDPF with SEVT thresholding by introducing a more expressive representation and leveraging non-linear manifold learning to uncover subtle, higher-order deviations in structural dynamics that linear models often fail to capture. The first stage of the proposed framework, Modal Feature Extraction, focuses on deriving vibration-based modal parameters using the SSI method. For each sensor channel, the PSD is estimated using Welch’s averaging technique [55], from which the dominant modal peaks are identified to form a modal frequency vector that characterises the resonant dynamics of the structure. In addition to modal parameters, the framework extracts a diverse set of enriched features to capture both global and localised vibration behaviour. Frequency-domain energies, obtained by integrating spectral power over predefined frequency bands, provide robustness against environmental and operational variability. Furthermore, cross-channel covariance features, derived from the covariance matrix and its eigenvalues, represent correlated vibration patterns across multiple sensors, reflecting spatial coherence in the structural response.

The second stage, feature normalisation, is a critical pre-processing step that ensures numerical stability, efficient convergence, and balanced feature contributions across both linear and non-linear modelling branches within the PCA–AE Framework. PCA, relying on covariance decomposition, and AE, leveraging gradient-based optimisation, a dual normalisation strategy is employed to accommodate their specific sensitivities. For the PCA branch, standardisation is applied to each feature to transform it into a zero-mean, unit-variance representation. For the non-linear AE branch, robust scaling or min–max normalisation [56] is adopted, depending on the presence of outliers and noise amplitude in the vibration signals. This normalisation enhances the gradient stability of the AE’s activation functions and ensures that all input features contribute proportionally during reconstruction error minimisation. PCA identifies the dominant axes of variance that represent the healthy-state vibration dynamics of the structure through SPE anomaly indicators. The corresponding SPE index [57] is computed as

(1) $$S_{PCA}=e^{T}e$$

where $e=x-\hat{x}$ is difference between data x and predicted data $\hat{x}$. A high SPE indicates structural anomalies, such as stiffness degradation, joint loosening, or changes in boundary conditions. The AE consists of an encoder–decoder pair. Once trained, the reconstruction error [58] for each new observation serves as its anomaly score (AE score), expressed as

(2) $$S_{AE}={\left||x-\hat{x}|\right|}_{2}^{2}$$

The mean squared error between the data x and the reconstructed data $\hat{x}$ Which is created by AE, gives the reconstruction error [58].

The third stage, combined with the normalised PCA and AE, provides a more comprehensive diagnostic perspective, enabling the framework to detect both subtle stiffness degradation and complex non-linear behaviours with enhanced reliability and precision across varying operational and environmental conditions. The overall fusion score is then computed as

(3) $$S_{fusion}=w_{PCA}S_{PCA}^{norm}+w_{AE}S_{AE}^{norm}$$

Subject to the constraint that the summation of weighted coefficients ($w_{PCA},w_{AE}$) is equal to one. The weighting coefficients are adaptively determined through performance-based weighting; the weights are determined based on validation-derived F1-scores [59], following $w_{PCA}{\;\propto F1}_{PCA\;}\;and\;w_{AE}\propto {F1}_{AE}$.

In the final stage of the ensemble fusion framework, the fused anomaly scores are analysed using an EVT–based approach to define a statistically reliable threshold [34] for damage detection. The parametric EVT approach, specifically the GPD, is tailored to model the statistical behaviour of rare, extreme deviations. In anomaly detection tasks, these rare deviations correspond to structural damage or abnormal behaviour, which manifest as tail events in the feature-score distribution. Unlike conventional methods that assume normality, EVT does not require the entire data set to be Gaussian; instead, it models tail exceedances, making it more appropriate for skewed, heavy-tailed, or otherwise non-Gaussian score distributions. The threshold is expressed as

(4) $$T_{fusion}=u+\frac{\sigma }{\xi }\left[{\left(1-p^{*}\right)}^{-\xi }-1\right]$$

where u is the threshold onset, σ is the scale parameter, ξ is the shape parameter, and p* Represents the chosen exceedance probability. This threshold effectively separates normal and abnormal structural states based on statistical tail behaviour. To improve durability under noisy conditions and environmental variability, a Bayesian smoothing step further refines $T_{fusion}$, producing a stable and adaptive decision boundary. Samples with fused scores exceeding this smoothed threshold are labelled as damaged, ensuring reliable and data-driven structural anomaly detection.

Algorithm 1 explains the AE–PCA Ensemble Fusion Anomaly Detection procedure used in this framework, illustrating how PCA and AE models are combined to capture both linear and non-linear structural behaviours. The performance of the proposed framework was rigorously evaluated under diverse operational and environmental conditions and compared with the CDPF-SEVT baseline using standard metrics, including Area Under the Curve (AUC), precision, recall, and F1 score.
**Algorithm 1.** AE–PCA Ensemble Fusion Anomaly Detection**Input:** Vibration segments {X_i}, i = 1, ..., N**Output:** Segment labels: Normal/Damaged1.Feature Extraction**For** each segment X_i:  f_i = [modal_features, time_features, frequency_features, covariance_features]2.Feature Scalingf_i_scaled = Standardize(f_i)f_i_PCA = PCA(f_i_scaled)f_i_AE = AE(f_i_scaled)3.Model TrainingTrain PCA_model on undamaged f_i_PCATrain AE_model on undamaged f_i_AE4.Compute Anomaly Scores**For** test segment f_i_test:  S_PCA = || f_i_test − PCA_model(f_i_test) ||_2  S_AE = || f_i_test − AE_model(f_i_test) ||_25.Normalisation and Fusion Score  S_PCA_norm = (S_PCA − μ_PCA)/σ_PCA  S_AE_norm = (S_AE − μ_AE)/σ_AE  S_fusion = α × S_PCA_norm + (1 − α) × S_AE_norm, 0 ≤ α ≤ 16.Threshold Estimation  Fit GPD on undamaged S_fusion  Determine smooth threshold T_smooth7.Segment Classification  **If** S_fusion > T_smooth → Label = Damaged  **Else** → Label = Normal

In summary, the Ensemble Fusion Framework significantly improves damage sensitivity, minimises false alarms, and ensures reliable generalisation across varying conditions. Its reliability and adaptability make it particularly well-suited for long-term monitoring of large-scale infrastructures, where damage often evolves gradually through a combination of linear and non-linear dynamic responses.

## 3. Case Study

### 3.1. Z24 Bridge

**Figure 2:**
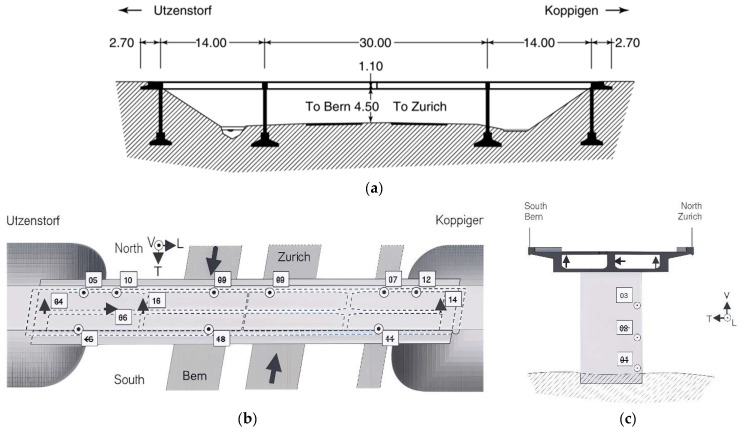
The Z24 Bridge: (**a**) the longitudinal section, (**b**) the top view, (**c**) the pier and girder cross-view.

The Z24 Bridge, an exemplary post-tensioned concrete box-girder structure, was built in Switzerland between 1961 and 1963. It was a significant engineering achievement of its time, characterised by its robust design and distinctive features. Illustrated in Figure 2, the bridge’s longitudinal section, main dimensions, top view, and pier cross-section provide a comprehensive overview of its structural layout. Despite being in relatively sound condition, the Z24 Bridge was ultimately demolished to pave the way for a new bridge designed to accommodate a larger side span, showcasing advancements in modern engineering approaches. Before the bridge was fully decommissioned, an extensive SHM program was initiated. This program aimed to assess the impact of environmental variability on the bridge by collecting detailed data, including acceleration time histories from strategically placed sensors. To obtain the acceleration time histories, sixteen accelerometers were attached to the bridge deck and the concrete column, and the location of these accelerometers is shown in Figure 2b,c. The sensor numbers struck through in Figure 2b,c failed during the operational period.

Understanding that environmental factors significantly affect structural dynamics, the SHM program employed five types of sensors to monitor critical variables. These included ambient temperature variations, air humidity levels, the occurrence of rainfall (true or false), wind speed, and wind direction. Among these, the influence of temperature was particularly emphasised, as it plays a crucial role in the structural behaviour of civil engineering projects. To accurately capture thermal data, temperature sensors were installed at eight specific points along the girder, located at the midpoint of the bridge’s three spans. Additionally, soil temperature measurements were taken adjacent to each concrete column, as well as at the north, central, and south sections of the intermediate piers, bringing the total number of sensors to 12.

In a progressive effort to simulate potential damage scenarios, the research team implemented controlled experiments on the bridge over the month preceding its demolition. These realistic scenarios were designed based on a series of progressive damage tests that included various structural impairments. Among these were the deliberate lowering and lifting of pier sections, intentional tilting of the foundation, and tests simulating concrete spalling at the soffit. Other scenarios involved induced abutment landslides, failures at concrete hinges, ruptures of anchor heads, and tendon failures. These tests were invaluable for understanding the bridge’s behaviour under stress and evaluating its structural integrity. Further details regarding the Z24 Bridge and the specific progressive damage scenarios can be referenced in [53], contained in the second panel.

**Figure 3:**
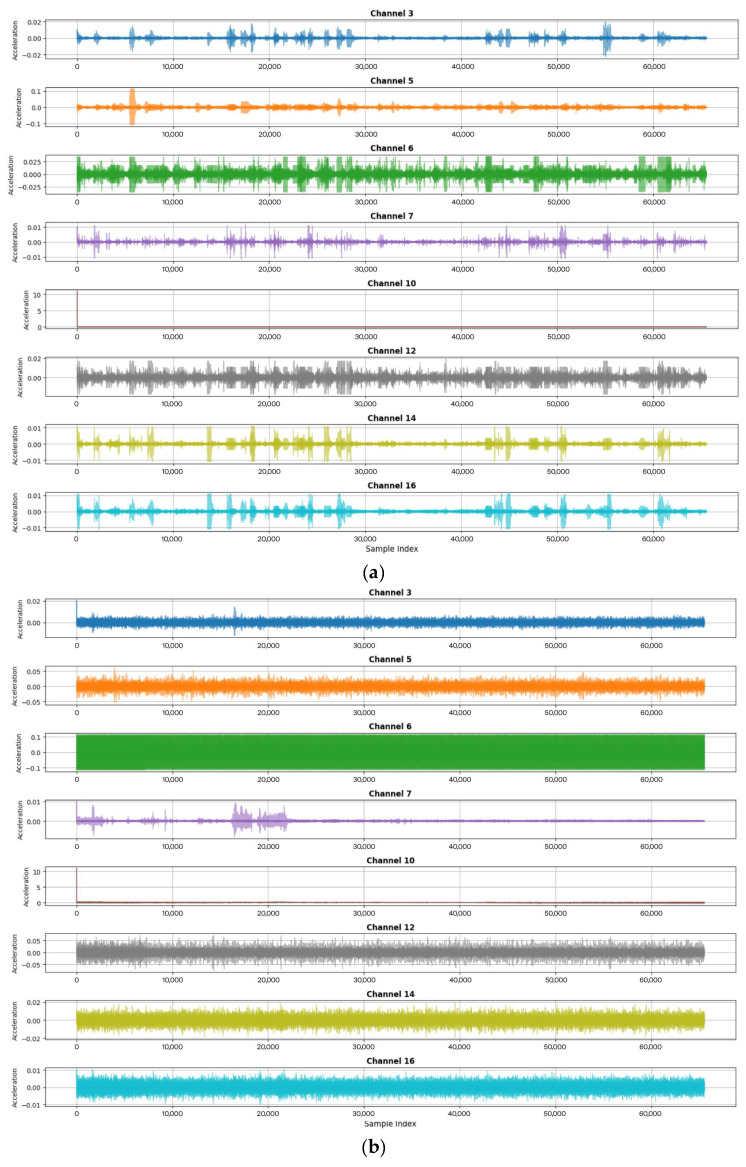
Dynamic response of the sensor in the Z24 bridge: (**a**) normal condition and (**b**) damaged condition.

Figure 3 illustrates the information gathered during a sub-measurement. The acceleration values at the designated measurement points were recorded continuously over time at a sampling frequency of 655 Hz (which equates to 655 samples per second). Through the application of operational modal analysis, specifically employing SSI, the modal frequencies of the bridge were identified across four modes for normal conditions and damaged conditions. Table 1 presents the modal frequencies corresponding to these modes, with a frequency difference ranging from 14 to 18 for the normal condition and 9 to 14 for the damaged condition.

The dataset used in this study consists of multichannel vibration measurements from the Z24 Bridge benchmark. For each monitoring timestamp, one .aaa ASCII file is provided per accelerometer channel, each containing 65,536 samples recorded at 100 Hz. Eight accelerometer channels were selected based on data completeness and their relevance to the global structural response. A segment is defined as a complete measurement instance in which all eight corresponding .aaa files are available. These files were time-aligned and combined into a synchronised multivariate matrix of size 65,536 × 8 (samples × channels). Across the available monitoring records, 48 segments representing the undamaged state and 48 segments representing the damaged state satisfied the completeness requirements and were therefore included in the analysis. These 96 multichannel segments serve as the basis for feature extraction, model training, and damage detection.

The undamaged segments were primarily used to train the baseline models and to establish feature normalisation for anomaly detection. For the proposed ensemble fusion model integrating PCA and AE features, a small fraction (≈5%) of damaged segments was included in the training set. This step, referred to as the semi-supervised phase, is an experimental procedure designed to enhance the model’s ability to generalise across the feature space and to stabilise the fusion of PCA and AE anomaly scores. Specifically, including only a very limited number of damaged examples allows the model to adapt slightly to feature variations near the decision boundary without significantly biasing the learned representation of normal, undamaged behaviour. This semi-supervised training approach is strictly for controlled experimental evaluation. In real-world SHM applications, it is generally not feasible to include damaged data during training, as anomalies are unknown a priori. The ensemble model is therefore fully compatible with operational deployment, where it would be trained exclusively on undamaged data and detect anomalies solely by deviations from learned normal patterns.

### 3.2. AUC Analysis

**Figure 4:**
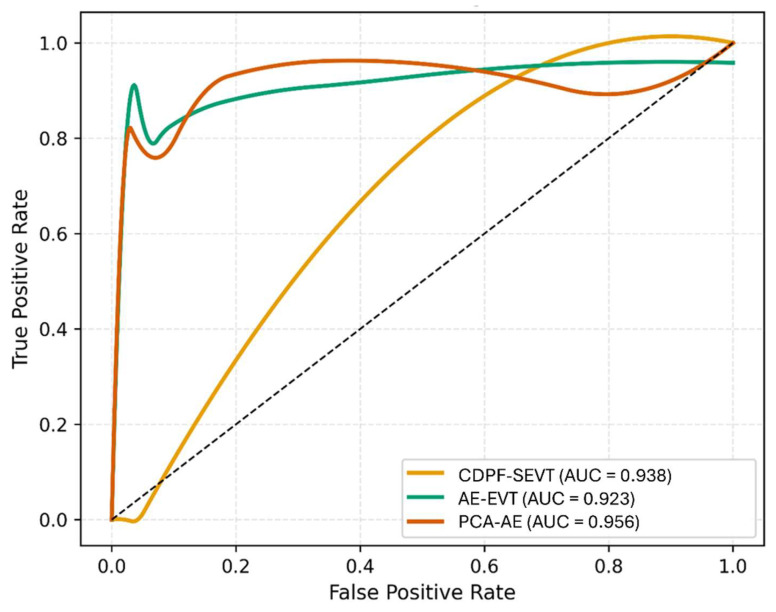
ROC curve comparison between baseline CDPF-SEVT, AE-EVT, and PCA-AE.

Figure 4 presents the Receiver Operating Characteristic (ROC) curves for the baseline CDPF–SEVT, AE–EVT, and the PCA–AE models. The ROC curve illustrates the trade-off between the True Positive Rate (TPR) and the False Positive Rate (FPR) at varying classification thresholds, providing a comprehensive view of each model’s detection sensitivity and false-alarm tendency. The AUC quantifies this discriminative capability, where higher AUC values correspond to improved distinction between damaged and undamaged structural conditions.

From the comparison, the baseline CDPF–SEVT model yields an AUC of 0.938, reflecting competent detection of linear modal shifts. The AE–EVT model exhibits comparable performance, leveraging non-linear feature representation to capture more complex response patterns. However, the PCA–AE framework attains the highest AUC of 0.956, signifying superior discriminative performance. This improvement results from the adaptive integration of PCA’s global variance sensitivity and the AE’s non-linear reconstruction capacity. The smooth and monotonic progression of the ROC curves indicates stable calibration and a well-balanced threshold response.

### 3.3. Classification Performance

**Figure 5:**
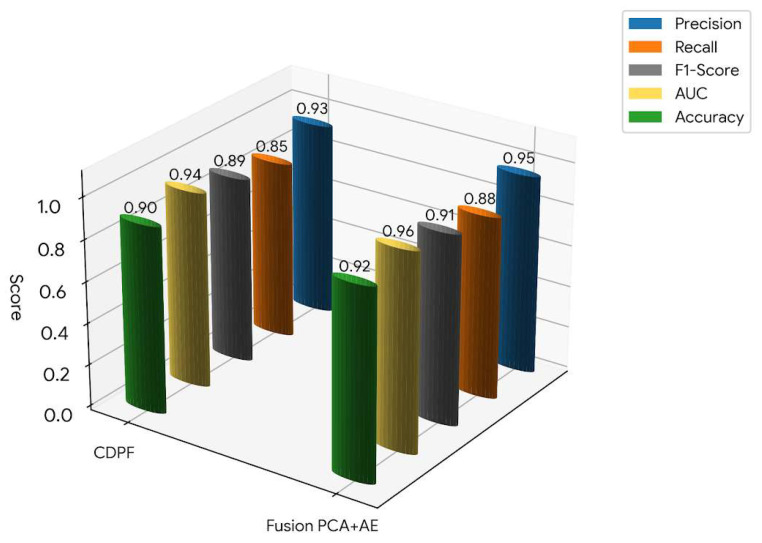
Performance metrics comparison between baseline CDPF and ensemble fusion PCA-AE.

The comparative performance metrics shown in Figure 5 illustrate Precision, Recall, F1-Score, and AUC for the baseline CDPF-SEVT and the PCA–AE models. These indices collectively assess the reliability, sensitivity, and overall classification accuracy, with classification used to evaluate detection performance, not the training procedure of the structural anomaly detection framework. The baseline CDPF-SEVT demonstrates balanced yet moderate performance (Precision = 0.93, Recall = 0.85, F1 = 0.89, AUC = 0.94), effectively capturing linear deviations but remaining limited in addressing non-linear response behaviours.

In contrast, the PCA–AE achieves consistently superior metrics (Precision = 0.95, Recall = 0.88, F1 = 0.91, AUC = 0.96), reflecting a synergistic balance between linear and non-linear feature spaces. Compared to the baseline CDPF-SEVT, the fusion approach enhances the F1-score by approximately 2.2%, indicating more reliable classification under varying structural and environmental conditions. Similarly, the AUC improvement of around 1.8% highlights the fusion model’s superior discrimination capability between healthy and damaged states. This improvement arises from the complementary fusion of PCA’s capacity to capture global modal shifts and the AE’s strength in modelling non-linear distortions. Such fusion-based approaches have been validated in recent literature, showing consistent gains in generalisation and reduced false-alarm rates for vibration-based SHM systems [60].

## 4. Results and Discussion

The discrimination performance between undamaged and damaged structural states, along with the adaptive weighting behaviour of the ensemble model, provides deeper insight into the dependability and interpretability of the proposed framework. The threshold-based score distributions clearly depict how the baseline CDPF-SEVT and PCA-AE models differentiate structural health conditions based on their respective anomaly indices. This complementary weighting mechanism ensures that the fusion process remains both sensitive to damage initiation and robust to environmental noise, thereby enhancing the overall reliability and interpretability of the anomaly detection framework. This adaptability is particularly important for long-term SHM, where environmental variability can easily mask damage-induced changes.

### 4.1. Threshold-Guided Structural State Classification

The discrimination between undamaged and damaged structural states is achieved through a statistically derived threshold-based anomaly detection framework. In this study, each model, the baseline CDPF-SEVT and the PCA–AE model, produces a continuous anomaly score that quantifies the deviation of current structural responses from their healthy-state characteristics. The threshold is parametrically determined using EVT by fitting a GPD to the tail of the undamaged score distribution, allowing adaptive decision boundaries that minimise false alarms from environmental or operational variability. For the baseline model, the anomaly scores are derived from the statistical energy of the reconstructed state-space residuals, effectively capturing the linear modal deviations. The Ensemble Fusion framework combines these linear and non-linear perspectives by integrating normalised PCA-based SPE scores and AE-based reconstruction errors through an adaptive weighted fusion mechanism.

**Figure 6:**
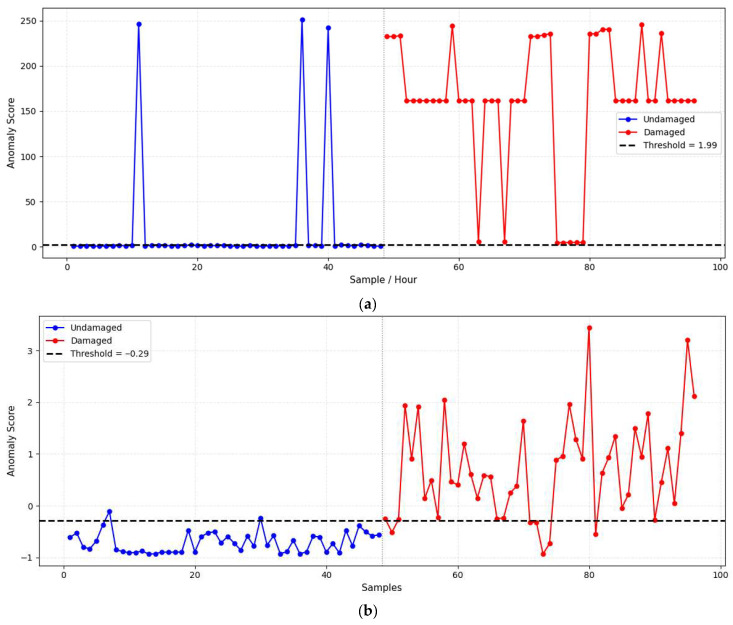
Anomaly detection based on threshold: (**a**) baseline CDPF-SEVT method, (**b**) ensemble fusion.

As illustrated in Figure 6, each method’s threshold distinctly separates the undamaged and damaged data clusters. The baseline CDPF-SEVT model shown in Figure 6a demonstrates moderate separation with some overlap near the threshold boundary, indicating limited sensitivity to non-linear distortions. However, the PCA–AE model produces the most distinct separation between classes, as shown in Figure 6b, with minimal overlap and well-defined threshold boundaries. It indicates enhanced sensitivity and durability, effectively capturing both global linear patterns and local non-linear irregularities in the structural response.

The clear separation observed between the undamaged and damaged clusters reflects the ensemble model’s superior discriminative capability and its potential for real-time SHM applications. By adaptively adjusting the threshold based on score distribution tails, the framework ensures reliable decision-making even under variable operating conditions.

### 4.2. Weighted Model Contribution Analysis

**Figure 7:**
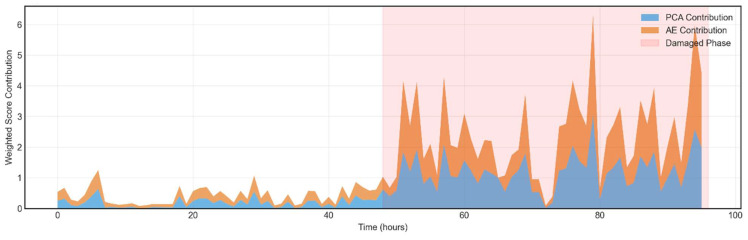
Stacked contribution area of PCA and AE for ensemble fusion.

**Figure 8:**
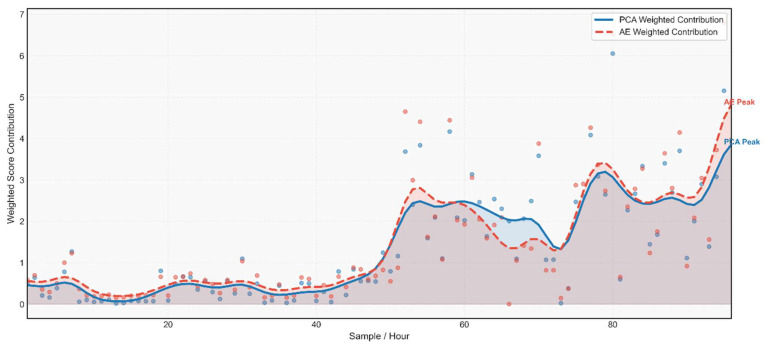
Weighted score contribution: PCA vs. AE.

The weighted score contribution analysis elucidates the relative influence of the PCA and AE components within the ensemble fusion framework. Since the proposed model integrates both linear and non-linear damage-sensitive features, an adaptive weighting mechanism is implemented to optimise their combined discriminative power. The normalised anomaly scores from PCA based on SPE and AE based on reconstruction error are fused to produce a unified damage index. Figure 7 and Figure 8 illustrate the normalised, weighted anomaly scores assigned by each model across all samples. Each plot represents the sample-wise influence of a specific model on the final ensemble decision.

The PCA Contribution plot, Figure 7, depicts the localised impact based on SPE. Peaks in the plot indicate samples where the PCA assigned comparatively higher anomaly scores, implying that the density structure of those observations deviates significantly from the global distribution. These instances correspond to periods where the structural behaviour becomes distinctly sparse or isolated in the feature space conditions often associated with abrupt or non-stationary responses. The AE Weighted Contribution Figure 8 illustrates the reconstruction-based anomaly scores, scaled according to the adaptive ensemble weight. Elevated values correspond to samples exhibiting dominant during subtle, non-linear anomalies.

Figure 8 illustrates the adaptive weighting contributions of the PCA and AE components within the Ensemble Fusion framework. The weighting mechanism governs the relative influence of each subsystem in the final fused score, ensuring that both linear and non-linear structural characteristics are appropriately represented. The weights are adaptively computed based on the statistical variance of the normalised anomaly scores, thereby emphasising the model that provides greater discriminatory information in each condition.

The presented results show that the PCA and AE modules contribute nearly equal weighting factors (≈0.5 each), indicating a balanced fusion between the two modalities. This equilibrium suggests that both global linear modal variations, captured by PCA, and localised non-linear distortions, captured by AE, play complementary roles in characterising structural behaviour. The adaptive weighting prevents dominance by either subsystem, thereby enhancing generalisation and mitigating overfitting. This balanced fusion mechanism aligns with recent advancements in hybrid anomaly detection frameworks, where score-level fusion and adaptive weighting techniques enhance the interpretability and reliability of SHM models under variable environmental and operational conditions.

### 4.3. Environmental and Operational Variability Analysis

Although long-term continuous monitoring data spanning multiple seasons would provide the most rigorous assessment of environmental and operational effects, such datasets with well-documented structural states are not always available in benchmark studies. In the context of unsupervised SHM, a widely adopted and practically meaningful alternative is to evaluate false-alarm behaviour using undamaged monitoring data. Any detected anomaly can be attributed to environmental or operational variability rather than structural damage. Following this rationale, the present study assesses the effectiveness of the proposed ensemble framework by analysing the distribution of anomaly scores and the false-alarm rate on undamaged segments of the Z24 dataset. This approach enables the quantification of the ensemble fusion mechanism’s impact on spurious detections. Indirect yet informative evidence of stability under the environmental and operational variability represented in the available data can thus be provided.

**Figure 9:**
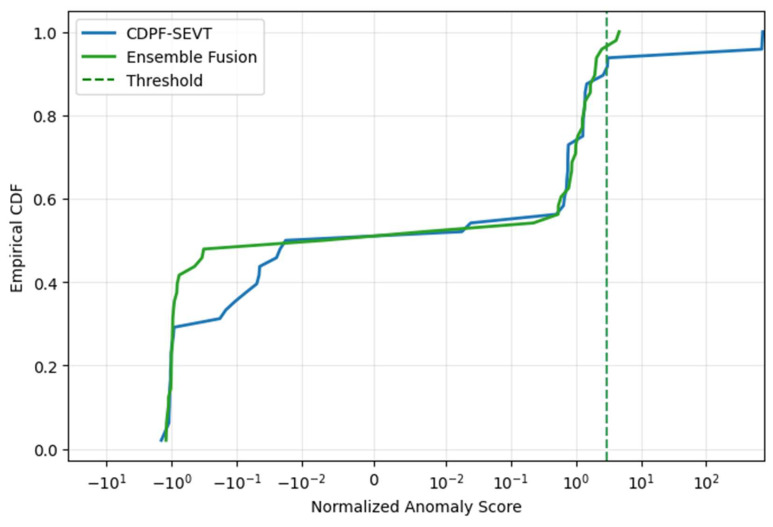
Empirical Cumulative Distribution Function of normalised anomaly scores for CDPF-SEVT and Ensemble Fusion methods.

**Figure 10:**
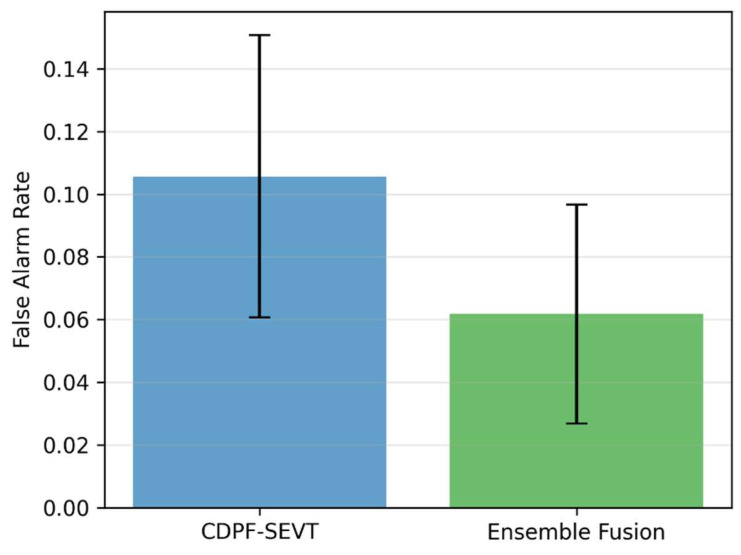
False Alarm Rate comparison between CDPF-SEVT and Ensemble Fusion methods.

The undamaged segments of the Z24 dataset were used to analyse and evaluate the effectiveness of the proposed ensemble fusion method under environmental and operational variability. The Empirical Cumulative Distribution Function (ECDF) of normalised anomaly scores (Figure 9) shows that the Ensemble Fusion method reduces the occurrence of extreme values compared to the baseline CDPF-SEVT method, indicating fewer spurious anomalies. The corresponding False Alarm Rate (FAR) analysis (Figure 10) confirms that Fusion maintains a low and stable FAR (~4%), whereas CDPF exhibits a higher and more variable FAR (~10%). These findings demonstrate that, within the environmental and operational variability represented in the dataset, the ensemble approach provides improved stability and reliability in anomaly detection.

A key limitation of the current study is the restricted environmental and operational variability represented in the Z24 dataset. The 48 undamaged and 48 damaged segments were collected over a relatively short monitoring interval, during which ambient temperatures varied only mildly, and no significant changes in operational loading (e.g., traffic intensity) were recorded. Consequently, frequency shifts due to temperature or operational effects were modest, and the dataset does not capture extreme environmental conditions such as seasonal freezing or high-traffic events. While the selected segments provide a well-defined benchmark with documented structural interventions, enabling methodological validation, future work will focus on evaluating the proposed ensemble framework on long-term continuous monitoring datasets that capture extreme conditions, including winter freezing periods, high-traffic events, and seasonal variations. This will enable a comprehensive assessment of false-alarm behaviour and robustness in real-world SHM scenarios, providing stronger validation for practical deployment.

## 5. Conclusions

This research presented an ensemble-based unsupervised anomaly detection framework for vibration-based Structural Health Monitoring (SHM), integrating linear and non-linear feature learning through Principal Component Analysis (PCA) and Autoencoder (AE) models and validated on a subset of the Z24 bridge benchmark dataset. Existing SHM approaches typically employ linear subspace models or nonlinear reconstruction-based techniques in isolation, relying on fixed ensemble fusion and thresholding strategies. In contrast, the proposed framework integrates complementary linear and nonlinear representations within a unified unsupervised setting. It further incorporates adaptive ensemble weighting and statistically principled threshold modelling.

The principal conclusions and quantitative findings are summarised as follows:

- The proposed PCA–AE ensemble fusion framework achieved superior detection performance with Precision = 0.95, Recall = 0.88, F1-score = 0.91, and AUC ≈ 0.956, demonstrating improved discrimination between undamaged and damaged structural states.
- Compared with the baseline CDPF–SEVT method (Precision = 0.93, Recall = 0.85, F1-score = 0.89, AUC = 0.94), the ensemble approach improved the F1-score by approximately 2.2% and the AUC by approximately 1.8%, indicating enhanced robustness and classification reliability.
- The adaptive score fusion strategy produced nearly balanced contributions from PCA and AE components (weights ≈ 0.5 each), confirming the complementary role of linear modal variation detection and non-linear response modelling in characterising structural behaviour.
- The EVT-based thresholding scheme enabled statistically principled decision boundaries without assuming score normality, resulting in improved stability of anomaly detection under the environmental and operational variability present in the dataset.
- False-alarm analysis on undamaged data used to analyse and evaluate the effectiveness of the proposed ensemble fusion method under environmental and operational variability demonstrated that the ensemble framework maintained a low and stable FAR of approximately 4%, compared to about 10% for the baseline CDPF–SEVT method, highlighting its improved resistance to spurious detections.

The results confirm that the proposed Ensemble Fusion PCA–AE framework offers enhanced accuracy, stability, and interpretability compared to conventional single-model approaches. While the present validation is limited by the restricted environmental and operational variability and the reduced number of segments available in the Z24 dataset, future work will focus on extending the framework to long-term continuous monitoring data, incorporating deeper ensemble architectures, and developing real-time adaptive weighting strategies to support robust deployment in real-world SHM applications.

## Figures and Tables

**Table 1:** Modal frequencies of the Z24 bridge based on OMA for normal conditions and damaged conditions.

Mode	Success Rate (%)	Eigenfrequency (Hz)					
		Normal Condition (Hz)		Damaged Condition (Hz)		Max. Difference (%)	
		Min	Max	Min	Max	Normal Condition	Damaged Condition
1	98	3.81	4.38	3.75	4.12	14	9
2	93	4.98	5.89	4.51	5.18	18	14
3	96	9.60	11.20	9.41	10.39	16	10
4	77	10.24	12.09	9.81	10.98	17	12

## Data Availability

The data used in this study are openly available and can be accessed from the following source: [57].
